# The Association of Isocaloric Substitution of Dietary Protein in Middle Age with Muscle Mass and Strength in Old Age: The Hordaland Health Study

**DOI:** 10.1016/j.cdnut.2023.102052

**Published:** 2023-11-30

**Authors:** Zoya Sabir, Anette Hjartåker, Jutta Dierkes, Hanne Rosendahl-Riise

**Affiliations:** 1 Center for Nutrition, Mohn Nutrition Research Laboratory, Department of Clinical Medicine, University of Bergen, Norway; 2 Department of Nutrition, Institute of Basic Medical Sciences, University of Oslo, Norway

**Keywords:** Protein, sarcopenia, muscle mass, muscle function, muscle health, aging

## Abstract

**Background:**

Age-associated loss of muscle mass and strength is an important predictor of disability in older persons. Although several mechanisms contribute to the decline in muscle mass and function seen with aging, the process is thought to be accelerated by an inadequate protein intake. However, the optimal amount and source of protein and the role of dietary protein intake over the life course remain uncertain.

**Objectives:**

In a sample of community-dwelling adults in Western Norway, the current study examined both cross-sectional and longitudinal associations over 20 y of dietary protein intake with appendicular skeletal muscle mass (ASMM) and muscle strength measured by handgrip strength (HGS) in older age.

**Methods:**

Dietary intake was assessed using food frequency questionnaires (FFQs) in middle age (46–49 y) and older age (67–70 y) within the community-based Hordaland Health Study.

**Results:**

Adjusted, multivariate linear regression analyses revealed a negative cross-sectional association between the substitution of total protein (TP) and animal protein (AP), with fat and carbohydrates, on ASMM in women but not in men. No longitudinal associations were found between substitution of dietary protein intake and ASMM in either sex in adjusted models. Similarly, no cross-sectional or longitudinal associations were evident between substitution of dietary protein intake and HGS in either sex in adjusted models.

**Conclusion:**

The findings in the current study highlight the need to clarify the role of dietary protein intake in the maintenance of muscle mass and muscle strength in healthy older adults.

## Introduction

The detrimental consequences of reduced muscle mass and strength with increasing age are well-documented [1]. Sarcopenia refers to the age-associated loss of muscle mass and strength beyond certain thresholds and remains among the major predictors of disability in older adults [2,3]. Decreased muscle mass and function have been associated with a multitude of chronic conditions, poor quality of life, and an increased risk of frailty and fractures [4]. Although several underlying mechanisms contribute to the decline in muscle mass and function seen with aging, the process may be accelerated by an inadequate dietary intake [5].

The official recommendation for protein intake of 0.83 g/kg bodyweight (bw)/d for adults (≥18 y) was reported by the FAO/WHO in 2007 [6]. Although the FAO/WHO recommendation is based on the lowest level of dietary protein intake that is required to balance obligatory nitrogen loss from the body and thereby maintain whole-body protein mass in healthy adults, it is unclear whether an increased dietary protein intake in older age may be protective against sarcopenia [7,8]. Notably, the WHO official recommendations for dietary protein have been challenged by independent expert groups. The Nordic Nutrition Recommendations 2023 (NNR2023) recommend a daily intake of 0.83 g/kg bw/d for adults and older adults based on nitrogen balance, whereas the recommended range of 1.2–1.5 g/kg bw/d for older adults (>70 y) is suggested to prevent decline of physical functioning [9]. The blunted postprandial muscle protein synthesis (MPS) in response to protein nutrition in older adults has been suggested to be a potential contributor to sarcopenia development, which may indicate that dietary protein requirements are increased in older adults [7,10]. However, findings from acute postprandial MPS studies predominantly using high-quality proteins cannot necessarily be translated to effects on relevant clinical parameters, such as skeletal muscle mass and function or whole-body protein balance, which forms the basis of the current protein recommendations [6,7,11].

Observational evidence about the role of protein intake in the maintenance of muscle mass and function is characterized by inconsistencies. For instance, a longitudinal analysis within the Framingham Third Generation Study (mean age 40±9 y) showed an association between lower dietary protein intake at baseline and lower appendicular lean mass (ALM) and quadriceps strength measured at follow-up 3–7 y later [12]. On the contrary, neither baseline protein intake nor change in protein intake were found to be associated with the risk of incident low grip strength over a 4-y follow-up period in middle-aged and older adults of the Korean Genome and Epidemiology Study [13]. Inconsistencies in findings across studies have also been addressed in a systematic review by Yaegashi et al. [14], which synthesized data from 17 observational studies on the association of protein intake with skeletal muscle mass in older adults (≥60 y). Significant positive associations were reported by 18 of the 26 available analyses, of which the majority were cross-sectional. Among the 9 cohort analyses, 5 found positive associations between protein intake and skeletal muscle mass [14]. Notably, in a recent meta-analysis of 9 longitudinal studies, Coelho-Junior et al. [15] reported no associations between protein intake and isometric handgrip strength (HGS) or walking speed in older adults.

Systematic reviews of intervention studies generally report that protein supplementation combined with exercise training increases lean mass, whereas studies using protein supplementation as their sole intervention have not demonstrated the same benefits [4,16]. Similarly, beneficial effects on muscle function have also been found for protein supplementation combined with exercise training interventions [4].

Besides the level of protein intake, the source or quality of protein has been suggested to be important to muscle mass and strength. Animal-based proteins are widely recognized as “high-quality” proteins due to their better digestibility, complete essential amino acid profile, and branched-chain amino acid content compared with plant-based proteins [17]. A meta-analysis of intervention studies [18] found no favorable effects of animal protein (AP) compared with plant protein (PP) on changes in lean mass or muscle strength in older adults, regardless of the inclusion of resistance exercise training (RET). However, a cross-sectional analysis within the NHANES showed that HGS increased with higher intakes of total protein (TP), AP, and PP, the increase being more prominent for AP [19]. Houston et al. [20] found that higher intakes of total and animal-based protein, but not plant-based protein, were associated with a lower decline in lean mass in older adults over a 3-y follow-up period. Whereas this may seem suggestive of a less favorable effect of plant-based protein compared with animal-based protein, various strategies have been proposed to overcome the seemingly lower anabolic properties of plant-based protein [7,21]. This remains particularly important regarding the environmental aspects of dietary protein intake [7].

Associations of dietary protein with muscle mass and strength have been investigated in relatively few population-based studies, most of which have been cross-sectional [14]. Additionally, the role of protein quality in the maintenance of muscle mass and strength remains largely unexplored. There has been a growing interest in the use of substitution modeling within the field of nutritional epidemiology [22]. Substitution modeling offers the opportunity to inform an optimal composition of the diet, and various substitution modeling methods have been used to investigate the effects of dietary components on health-related outcomes [22,23]. The general concept of isocaloric substitution is to determine the impact on a specific outcome when replacing the intake of a given dietary component with another calorically comparable dietary component [23]. The current study implemented isocaloric substitution modeling to study the associations of substituting TP and AP intake with muscle mass and muscle strength.

As loss of muscle mass and strength is known to be a gradual process, there is an inevitable need for longitudinal studies with more extensive follow-up durations. In a sample of community-dwelling older adults in Norway, the current study, hence, aimed to examine the following: *1*) cross-sectional associations of dietary protein intake with muscle mass and muscle strength in older age, and *2*) longitudinal associations of dietary protein intake in middle age with muscle mass and muscle strength in older age (follow-up duration of ∼20 y).

## Methods

### Ethical approval

The current study was conducted following the principles laid down in the Declaration of Helsinki, and all procedures involving humans, including dietary data collection, were approved by the Regional Committee for Medical and Health Research Ethics West (HUSK2 REK 2009/825, HUSK3 REK 2017/294). All participants provided written informed consent. Participation was voluntary, and withdrawal was possible at any time without further justification.

### Study sample

The current study used data from the Hordaland Health Study (HUSK), a community-based observational study in Western Norway. HUSK comprises 3 health surveys conducted between 1992 and 2020, in which specific birth cohorts and random samples of the population were invited to participate. Residents of Hordaland County (currently part of Vestland County) born between 1925 and 1927 or 1950 and 1952 were invited to participate in the first survey, HUSK1 (1992–1993). In the second survey, HUSK2 (1997–1999), previous participants from the 1925–1927 and 1950–1951 birth cohorts were reinvited. Finally, participants born from 1950 to 1951 who had taken part in both HUSK1 and HUSK2 were reinvited to the third study wave, HUSK3 (2018–2020). The present analysis includes data from HUSK2 and HUSK3, both of which estimated habitual dietary intake by food frequency questionnaires (FFQs). Additional information may be found on the official HUSK webpage (https://husk-en.w.uib.no/).

In the 1997–1999 study, all living participants born from 1950 to 1951 and residing in Bergen or the neighboring municipalities were reinvited (*n* = 4849) to participate in HUSK2. The survey included a brief health examination and collection of nonfasting blood samples. Plasma cotinine concentration was used as a marker of recent nicotine use, which allowed objective identification of current smokers [24]. Self-administered questionnaires were used to collect information on lifestyle characteristics, and a semiquantitative paper-based FFQ (PB-FFQ) was used to estimate dietary intake.

In HUSK3 (2018–2020), all men and women born in 1950–1951 who had previously participated in HUSK1 and HUSK2 were reinvited. Eligible subjects received the invitation by mail. Electronic informed consent was signed by 2252 participants, out of which 2183 attended the health survey. The health survey included blood pressure measurements, body composition assessment, echocardiography, blood sample collection, and HGS measurement. Information about lifestyle characteristics was obtained through a self-administered electronic questionnaire. Dietary intake was assessed by a web-based FFQ (WebFFQ).

**FIGURE 1:**
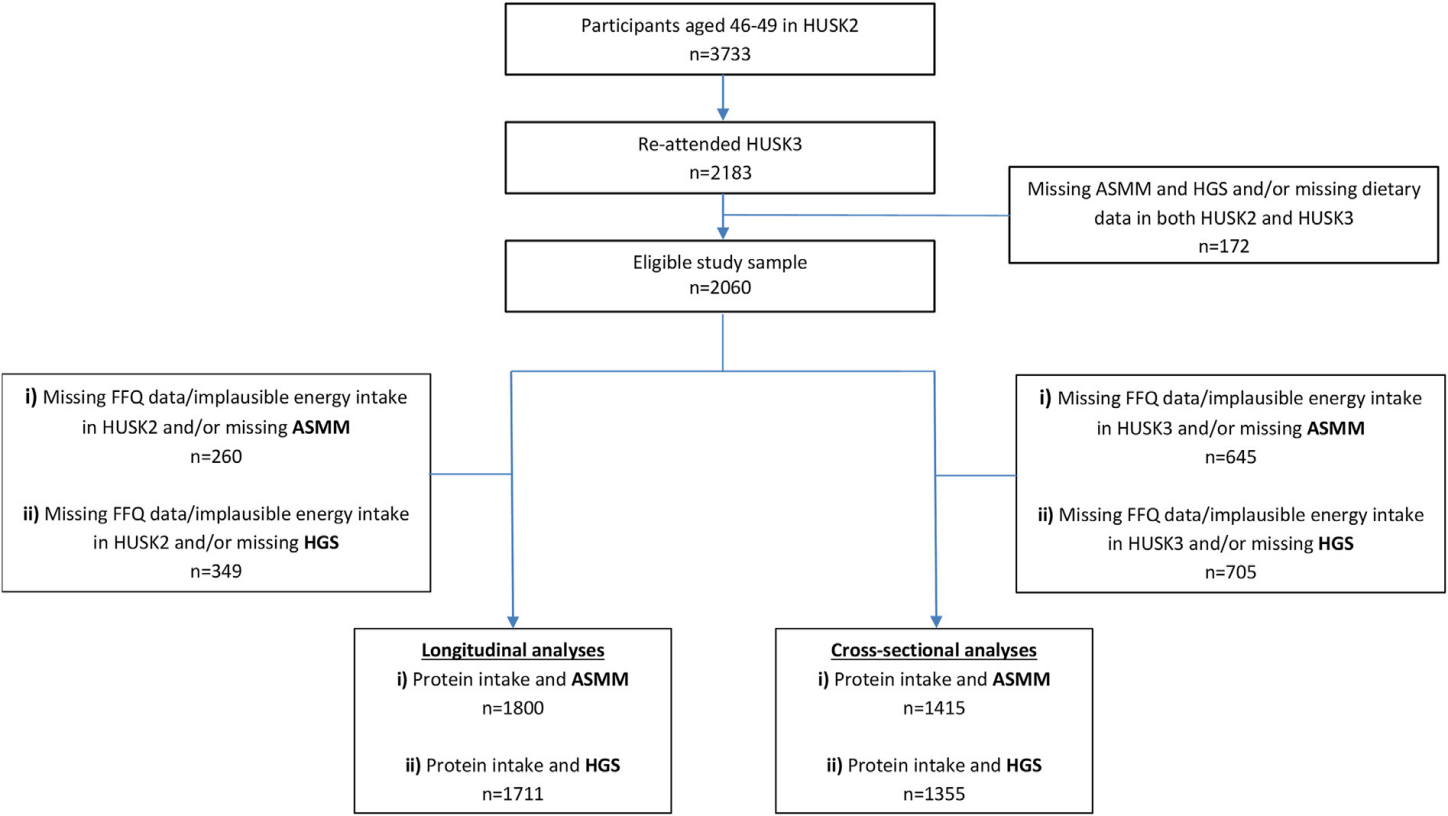
Flowchart illustrating selection of participants from the Hordaland Health Study for cross-sectional and longitudinal analyses. ASMM, appendicular skeletal muscle mass; FFQ, food frequency questionnaire; HGS, handgrip strength.

The present study includes only males and females who participated in both HUSK2 (age 47–49 y) and HUSK3 (age 67–70 y). Participants with missing FFQ data in both HUSK2 and HUSK3 were excluded. Further, participants with missing data on both study outcomes, i.e., muscle mass and muscle strength in HUSK3, were excluded. The main analytical sample was, hence, comprised of 2060 participants eligible for statistical analyses. Participants with implausible estimates of energy intake in HUSK2 were excluded before longitudinal regression analyses. In contrast, participants with implausible estimates of energy intake in HUSK3 were excluded from the cross-sectional regression analyses. Implausible energy intake estimates were defined as <3300 kJ/d or >17,500 kJ/d for men and <3000 kJ/d or >15,000 kJ/d for women (Figure 1). In the current study, associations of dietary protein intake with muscle mass and muscle strength were studied using regression models based on isocaloric substitution of macronutrients.

### Dietary intake assessment in HUSK2 and HUSK3

Dietary intake in HUSK2 was assessed using a PB-FFQ, which is a modified version of an FFQ developed at the Department of Nutrition, University of Oslo [[25], [26], [27]]. The PB-FFQ is a previously validated 169-item dietary assessment tool that aimed to collect information about habitual dietary intake during the past year [[25], [26], [27]]. The PB-FFQ was completed by 87% of the participants, corresponding to 3107 men and women aged 46–49 y. In HUSK3, habitual food and nutrient intake was estimated by a 279-item WebFFQ developed at the Department of Nutrition, University of Oslo. The WebFFQ is a further development of the PB-FFQ previously administered in HUSK2, allowing the inclusion of food items that were not previously available. Adequate relative validity of the WebFFQ has been demonstrated within a subpopulation of HUSK3 [28]. The WebFFQ was completed by 78% of the attending participants, corresponding to 1704 men and women aged 67–70 y.

Daily intakes of energy and nutrients in both HUSK2 and HUSK3 were estimated by a software system and its corresponding food databases developed by the Department of Nutrition, University of Oslo (HUSK2: KBS version 3.2, database IE-96; HUSK3: KBS version 7.4, database AE-18). The IE-96 and AE-18 databases are extended versions of the official Norwegian Food Composition Table [29]. Dietary supplement intake was included in the nutrient calculations.

### Categorization of protein sources

Total daily protein intake in both HUSK2 and HUSK3 was grouped into 3 main categories: AP, PP, and unspecified protein (UP). The category of AP was comprised of the following foods: meat and meat products, fish and fish products, shellfish, egg, milk and milk products, cheese, and butter. AP was further divided into marine protein consisting of fish and shellfish, as well as dairy protein consisting of milk and milk products, cheese, and butter. The category of PP consisted of bread, flour, rice, pasta, breakfast cereals, potatoes (including potato chips), vegetables, fruits and berries, margarine, coffee, and tea. Mixed dishes and products for which source of protein could not be determined clearly, e.g., cakes, mayonnaise, dressings, and sauces, were classified as UP sources. TP intake, as well as source-specific protein intake, were expressed as grams per d (g/d), grams per megajoule (g/MJ), grams per kilogram/bw (g/kg bw), and energy percentage (E%).

### Measurement of outcomes

#### Handgrip strength

Muscle strength, represented by HGS, was measured in HUSK3 using a Jamar+ Digital Hand Dynamometer. Three measurements were performed on each hand, alternating between the left and right hand after each measurement. When performing the measurement, participants were seated with feet flat on the ground, shoulders adducted, the elbow flexed at a 90° angle, and the forearm and wrist in a neutral position. Participants were instructed to squeeze the grip handle of the dynamometer with maximum effort for 3–5 sec, while trying to keep the rest of the body as still as possible. HGS was expressed in kilograms (kgs), and the current study applied the highest recorded measurement (MaxHGS) in the statistical analyses. The test was conducted by a trained dietitian. Participants with medical conditions or experiencing pain affecting the shoulder, forearm, wrist, or hand, were not tested for HGS.

#### Appendicular skeletal muscle mass

Skeletal muscle mass was determined by bioelectrical impedance analysis (BIA) using the SECA mBCA 515 (Seca). The measurement was performed by instructing participants to stand bare feet on the foot electrodes located on the device platform. Participants were then requested to lightly grip a pair of hand electrodes on the handrail, allowing their arms to be extended without strain. Once the machine detected sufficient contact with all 4 electrodes, the measurement was initiated. Following completion of the BIA measurement, activity level, measured waist circumference (cm) and measured height (cm) were entered into the device. Appendicular skeletal muscle mass (ASMM) (kgs) was calculated by entering BIA-derived estimates of resistance (R) and reactance (Xc) into the equation by Kyle et al. [30].

### Assessment of smoking and physical activity

The variable for current smoking (yes/no) in HUSK3 was based on self-reported questionnaire data. In HUSK2, nonfasting blood samples were collected. Cotinine was measured in EDTA plasma stored at −80°C until analyzed at Bevital A/S (www.bevital.no) by LC/MS/MS. Participants with plasma cotinine concentrations of ≥85 nmol/L were classified as current smokers. For participants with missing cotinine measures in HUSK2, self-reported smoking status was applied. Physical activity assessment in HUSK2 and HUSK3 was done through questionnaire items that inquired about how many hours of light/moderate and hard physical activity participants completed per week. The questionnaire items specified that light/moderate physical activity included any activity that did not result in sweating or shortness of breath, whereas hard physical activity included any activity that would result in sweating or shortness of breath. The variable for self-reported physical activity was categorized into yes/no in both HUSK2 and HUSK3. At each time point, participants were classified as physically active if reporting any amount of hard physical activity or ≥3 h of light/moderate physical activity per week.

### Statistical analysis

Distribution of participant characteristic variables and dietary intake variables was assessed by visual inspection of histograms and Q-Q plots. Participant characteristics are reported as means (±SD) for normally distributed continuous variables and as proportions for categorical variables. For continuous variables, the independent samples t-test was applied to test for sex differences in characteristics. For categorical variables, Pearson’s chi-squared test was applied to test for sex differences in characteristics (*P* values not shown).

Multivariate linear regression analyses with substitution of macronutrients were conducted for men and women separately to study the following: *1*) cross-sectional associations of dietary protein intake (total and animal compared with plant) with muscle mass and muscle strength in older age and *2)* longitudinal associations of dietary protein intake (total and animal compared with plant) in middle age (HUSK2) with muscle mass and muscle strength in older age (HUSK3). Substitution models accounting for the confounding effects of total energy intake and other macronutrients were created. Energy percentages of all energy-providing nutrients (fat, carbohydrate, and alcohol), with the exception of the macronutrient being studied (TP/AP), were simultaneously entered into the model. The inclusion of the 3 mentioned macronutrients in the substitution model allowed the estimation of all relevant contrasts between macronutrient effects. The estimated regression coefficient (B) for each macronutrient derived from the linear regression analysis represents the effect on the outcome variable (ASMM or HGS) when increasing the intake of the macronutrient in question by 1E% at the expense of the excluded nutrient, while holding the intake of the other macronutrients and energy constant. Hence, our model tests the effect of substituting TP with fat or carbohydrates and substituting AP with PP, fat, or carbohydrates, while holding energy intake constant, on ASMM and HGS. Cross-sectional regression models were adjusted for potential confounders obtained in HUSK3 and included BMI (kg/m^2^), smoking status (yes/no), and physical activity (inactive/active). Longitudinal regression models were adjusted for potential confounders obtained in HUSK2 and included BMI (kg/m^2^), smoking status (yes/no), and physical activity (inactive/active). All *P* values presented are 2-sided. *P* values <0.05 were considered statistically significant. Statistical analyses were conducted in Statistical Package for Social Sciences version 28, IBM Corporation (IBM SPSS Statistics for Windows, version 28.0. IBM Corp; 2021).

## Results

### Participant characteristics

Characteristics of the 2060 participants eligible for statistical analysis are presented in Table 1. BMI did not differ significantly between men and women. The updated consensus recommendations by the European Working Group for Sarcopenia in Older People (EWGSOP2) define low HGS by a cut-off of <27 kg in men and <16 kg in women, whereas low ASMM is defined by values of <20 kg in men and <15 kg in women. Mean values of MaxHGS (kg) and ASMM (kg) exceeded the EWGSOP2 cut-offs for low muscle strength and muscle mass, respectively [5]. The proportion of men and women with low HGS was 1.1% and 1.8%, respectively. Low ASMM was evident in a considerably higher proportion of women than men. Although the percentage of men and women who reported ≥3 hours of moderate leisure time physical activity per week were similar, a greater percentage of men than women engaged in hard physical activity weekly.

**TABLE 1:** Characteristics of participants eligible for statistical analysis (*n* = 2060) in HUSK3

Characteristics^a^	All (*n* = 2060)	Men (*n* = 924)	Women (*n* = 1136)
Height (cm)	171 (9.2)	179 (6.1)	165 (6.0)∗
Weight (kg)	77.2 (14.5)	85.5 (12.3)	70.5 (12.5)∗
BMI (kg/m^2^)	26.4 (4.1)	26.8 (3.5)	26.1 (4.5)∗
Waist (cm)	95.2 (12.6)	100.5 (10.7)	91.0 (12.4)∗
Total body fat mass (kg)	27.3 (8.8)	25.2 (8.1)	29.1 (8.8)∗
Total body fat mass percentage (%)	35.3 (8.3)	28.8 (6.0)	40.4 (6.0)∗
Maximum HGS (kg)	34.6 (10.8)	44.6 (7.1)	26.3 (4.6)∗
Low HGS (%)^b^	1.5	1.1	1.8
ASMM (kg)	19.4 (4.6)	23.8 (2.6)	15.9 (2.2)∗
Low ASMM (%)^b^	22.7	5.2	36.6∗
Regular, current smoking^c^	7.8	7.3	8.3
Education >12 y^c^^,^^d^	45.6	50.1	42.0∗
Leisure time moderate physical activity^c^			
≥3 h per wk (%)	70.2	70.6	69.8
Leisure time hard physical activity^c^			
Any (%)	86.9	90.8	83.8∗
Marital status^c^^,^^d^			
Married (%)	78.7	82.0	76.1∗
Medical conditions^c^(%)			
Diabetes	6.3	8.5	4.5∗
COPD	4.0	4.4	3.7
Cancer	2.7	4.4	1.3∗
Osteoporosis	13.1	6.3	18.6∗
Rheumatoid arthritis	3.3	3.6	3.0∗

Abbreviations: ASMM, appendicular skeletal muscle mass; BMI, body mass index; COPD, chronic obstructive pulmonary disease; HGS, handgrip strength. Slight deviations in *n* for some participant characteristics due to lack of data.

∗ Statistically significant difference between men and women tested by independent samples t-test for continuous variables and chi-square/Fisher exact test for categorical variables, significance level of *P* < 0.05.

a Continuous variables are presented as mean±standard deviation. Categorical variables are presented as percentages.

b Low HGS: <27 kg in men, <16 kg in women; Low ASMM: <20 kg in men, <15 kg in women.

c Based on self-reported data.

d Characteristic obtained in HUSK2.

#### Dietary protein intake

Estimated mean daily intakes of energy, TP, AP, and PP for all participants, as well as for men and women separately, are presented in Table 2. Higher mean daily intakes of TP, AP, and PP were evident in HUSK3 than in HUSK2, regardless of whether intake was expressed in absolute or relative units. Men had consistently higher absolute daily intakes (g/d) of TP, AP, and PP than women in both HUSK2 and HUSK3. Relative daily intakes of dietary protein expressed as g/MJ, E%, and g/kg bw were generally higher in women than in men.

**TABLE 2:** Daily nutrient intake of eligible study participants (*n* = 2060) in HUSK2 and HUSK3.

	All (*n* = 2060)	Men (*n* = 924)	Women (*n* = 1136)	All (*n* = 2060)	Men (*n* = 924)	Women (*n* = 1136)
	Mean (95% CI)	Mean (95% CI)	Mean (95% CI)	Mean (95% CI)	Mean (95% CI)	Mean (95% CI)
	HUSK2			HUSK3		
Energy, MJ	9.1 (9.0, 9.2)	10.5 (10.3-10.7)	8.0 (7.9-8.2) †	11.3 (11.0-11.5)∗	12.3 (12.1-12.6)	10.4 (10.1-10.7) †
Protein, g/d	85.2 (84.0, 86.4)	96.7 (94.9-98.5)	76.2 (74.9-77.6) †	120.1 (117.9-122.4)∗	131.4 (128.1-134.6)	111.0 (108.1-113.9) †
Protein, g/MJ	9.4 (9.4, 9.5)	9.2 (9.2-9.3)	9.6 (9.5-9.6) †	10.8 (10.8-10.9)∗	10.7 (10.6-10.9)	10.9 (10.8-11.0) †
E%	16.0 (15.9, 16.1)	15.7 (15.6-15.8)	16.3 (16.1-16.4) †	18.4 (18.3-18.6)∗	18.3 (18.1-18.5)	18.6 (18.4-18.7) †
Protein, g/kg bw	1.16 (1.14, 1.17)	1.17 (1.14, 1.19)	1.15 (1.12, 1.17)	1.60 (1.57, 1.63)∗	1.56 (1.52, 1.60)	1.62 (1.58, 1.67)
Animal protein, g/d	51.4 (50.5, 52.3)	58.2 (56.8, 59.6)	46.1 (45.1, 47.1) †	76.7 (75.1, 78.2)∗	85.0 (82.6, 87.4)	69.9 (68.0, 71.8) †
Marine, g/d	12.8 (12.5, 13.2)	14.2 (13.6, 14.8)	11.8 (11.3, 12.3) †	26.4 (25.6, 27.2)∗	29.7 (28.4, 31.0)	23.8 (22.8, 24.7) †
Dairy, g/d	18.2 (17.7, 18.7)	20.4 (19.6, 21.1)	16.5 (15.9, 17.1) †	19.6 (18.9, 20.3)∗	20.2 (19.2, 21.2)	19.1 (18.2, 20.0)
Animal protein, g/MJ	5.7 (5.6, 5.7)	5.6 (5.5, 5.6)	5.8 (5.7, 5.9) †	7.0 (6.9, 7.1)∗	7.0 (6.9, 7.1)	7.0 (6.9, 7.1)
Marine, g/MJ	1.4 (1.4, 1.5)	1.4 (1.3, 1.4)	1.5 (1.4, 1.5) †	2.4 (2.4, 2.5)∗	2.5 (2.4, 2.6)	2.4 (2.3, 2.5)
Dairy, g/MJ	2.0 (2.0, 2.1)	2.0 (1.9, 2.0)	2.1 (2.0, 2.1) †	1.8 (1.7, 1.8)∗	1.7 (1.6, 1.7)	1.9 (1.8, 2.0) †
Animal protein, g/kg bw	0.70 (0.68, 0.71)	0.70 (0.68, 0.72)	0.69 (0.68, 0.71)	1.02 (0.99, 1.04)∗	1.01 (0.98, 1.04)	1.02 (0.99, 1.05)
Marine, g/kg bw	0.17 (0.17, 0.18)	0.17 (0.16, 0.18)	0.18 (0.17, 0.18)	0.35 (0.34, 0.36)∗	0.35 (0.34, 0.37)	0.35 (0.33, 0.36)
Dairy, g/kg bw	0.25 (0.24, 0.25)	0.25 (0.24, 0.25)	0.25 (0.24, 0.26)	0.26 (0.25, 0.27)∗	0.24 (0.23, 0.25)	0.28 (0.27, 0.30) †
Animal protein, E%	9.5 (9.4, 9.6)	9.3 (9.1, 9.4)	9.7 (9.5, 9.8) †	11.7 (11.6, 11.9)∗	11.7 (11.5, 12.0)	11.7 (11.5, 11.9)
Plant protein, g/d	29.8 (29.4, 30.3)	34.0 (33.3, 34.7)	26.6 (26.1, 27.0) †	39.3 (38.4, 40.3)∗	41.6 (40.3, 42.8)	37.5 (36.2, 38.9) †
Plant protein, g/MJ	3.3 (3.3, 3.4)	3.3 (3.2, 3.3)	3.4 (3.3, 3.4) †	3.5 (3.4, 3.5)∗	3.4 (3.3, 3.4)	3.6 (3.5, 3.6) †
E%	5.6 (5.5, 5.6)	5.5 (5.4, 5.5)	5.6 (5.6, 5.7) †	5.8 (5.8, 5.9)∗	5.6 (5.5, 5.7)	6.0 (5.9, 6.1) †
Plant protein, g/kg bw	0.41 (0.40, 0.41)	0.41 (0.40, 0.42)	0.40 (0.39, 0.41) †	0.53 (0.51, 0.54)∗	0.50 (0.48, 0.51)	0.55 (0.53, 0.57) †

Abbreviations: bw, body weight; CI, confidence interval; E%, proportion of total energy from protein; MJ, megajoule.

∗ Statistically significantly different from mean values of dietary intake in HUSK2 for total group (*n* = 2060) tested by paired samples t-test, significance level *P* < 0.05.

† Statistically significant difference in dietary intake between men and women within the given study wave tested by independent samples t-test, significance level *P* < 0.05.

#### Cross-sectional relationship of protein intake with ASMM and HGS

Results from multivariate linear regression analyses are presented in Table 3. Unadjusted cross-sectional analysis of dietary protein intake in HUSK3 and ASMM in HUSK3 showed a statistically significantly lower ASMM when substituting 1E% of TP with fat or carbohydrates in both men and women. Further, substituting 1E% AP with fat or carbohydrates showed a statistically significantly lower ASMM in both men and women, whereas substitution of AP with PP was not associated with ASMM in either sex. Following adjustment for HUSK3 covariates, the associations of substituting TP and AP (with fat or carbohydrates) and ASMM remained statistically significant in women but not in men. No associations between substituting TP (with fat or carbohydrates) or AP (with PP, fat, or carbohydrates) and HGS were statistically significant in unadjusted or adjusted cross-sectional analyses in either sex.

**TABLE 3:** Sex-specific cross-sectional and longitudinal associations of amount and source of protein with ASMM and HGS in the Hordaland Health Study. Estimates obtained from multiple linear regression analysis with substitution of macronutrients (E%).

	Appendicular skeletal muscle mass (kg)				Handgrip strength (kg)			
	Cross-sectional associations							
	Men (*n* = 632)		Women (*n* = 783)		Men (*n* = 621)		Women (*n* = 734)	
	Estimate (95% CI)	*P* value	Estimate (95% CI)	*P* value	Estimate (95% CI)	*P* value	Estimate (95% CI)	*P* value
Total protein								
Substituted by fat								
M1	-0.099 (-0.175, 0.023)	0.011	-0.094 (-0.148, -0.040)	<0.001	-0.048 (-0.259, 0.162)	0.651	0.018 (-0.101, 0.136)	0.770
M2^a^	-0.012 (-0.068, 0.044)	0.672	-0.042 (-0.080, -0.004)	0.032	-0.035 (-0.247, 0.177)	0.746	0.030 (-0.091, 0.150)	0.627
Substituted by carbohydrates								
M1	-0.090 (-0.160, -0.020)	0.012	-0.111 (-0.162, -0.061)	<0.001	-0.061 (-0.259, 0.138)	0.550	-0.059 (-0.170, 0.052)	0.295
M1^a^	0.000 (-0.051, 0.052)	0.986	-0.046 (-0.083, -0.010)	0.012	-0.059 (-0.260, 0.142)	0.563	-0.045 (-0.158, 0.068)	0.436
Animal protein								
Substituted by plant protein								
M1	-0.119 (-0.309, 0.070)	0.217	-0.015 (-0.137, 0.107)	0.806	0.096 (-0.430, 0.623)	0.720	0.129 (-0.141, 0.399)	0.348
M2^a^	0.006 (-0.133, 0.144)	0.933	0.029 (-0.057, 0.115)	0.508	0.068 (-0.461, 0.598)	0.800	0.131 (-0.139, 0.402)	0.341
Substituted by fat								
M1	-0.111 (-0.188, -0.033)	0.005	-0.106 (-0.161, -0.051)	<0.001	-0.022 (-0.238, 0.194)	0.840	0.021 (-0.100, 0.142)	0.732
M2^a^	-0.017 (-0.074, 0.040)	0.564	-0.043 (-0.082, -0.004)	0.031	-0.012 (-0.230, 0.206)	0.914	0.033 (-0.090, 0.156)	0.594
Substituted by carbohydrates								
M1	-0.094 (-0.166, -0.022)	0.010	-0.125 (-0.177, -0.074)	<0.001	-0.040 (-0.243, 0.162)	0.696	-0.059 (-0.172, 0.055)	0.311
M2^a^	-0.005 (-0.058, 0.048)	0.855	-0.049 (-0.086, -0.012)	0.010	-0.040 (-0.245, 0.165)	0.701	-0.045 (-0.161, 0.072)	0.453
Longitudinal associations								
	Men (*n* = 770)		Women (*n* = 1030)		Men (*n* = 751)		Women (*n* = 960)	
	Estimate (95% CI)	*P*	Estimate (95% CI)	*P*	Estimate (95% CI)	*P*	Estimate (95% CI)	*P*
Total protein								
Substituted by fat								
M1	-0.141 (-0.238, -0.045)	0.004	-0.058 (-0.122, 0.006)	0.073	-0.144 (-0.421, 0.133)	0.307	-0.162 (-0.301, -0.024)	0.021
M2^b^	-0.009 (-0.091, 0.073)	0.828	0.042 (-0.009, 0.093)	0.109	-0.108 (-0.389, 0.173)	0.450	-0.116 (-0.255, 0.024)	0.103
Substituted by carbohydrates								
M1	-0.174 (-0.264, -0.085)	<0.001	-0.038 (-0.095, 0.020)	0.203	-0.117 (-0.372, 0.138)	0.367	-0.121 (-0.246, 0.005)	0.060
M2^b^	-0.036 (-0.112, 0.040)	0.355	0.045 (-0.001, 0.091)	0.056	-0.091 (-0.351, 0.169)	0.492	-0.104 (-0.230, 0.022)	0.105
Animal protein								
Substituted by plant protein								
M1	-0.244 (-0.455, -0.032)	0.024	0.049 (-0.099, 0.197)	0.515	-0.210 (-0.837, 0.417)	0.510	0.091 (-0.234, 0.417)	0.582
M2^b^	0.019 (-0.160, 0.197)	0.837	0.047 (-0.070, 0.164)	0.429	-0.167 (-0.801, 0.467)	0.604	0.062 (-0.260, 0.385)	0.705
Substituted by fat								
M1	-0.168 (-0.267, -0.069)	P<0.001	-0.096 (-0.163, -0.029)	0.005	-0.167 (-0.453, 0.119)	0.252	-0.178 (-0.324, -0.031)	0.018
M2^b^	-0.015 (-0.099, 0.069)	0.723	0.023 (-0.031, 0.077)	0.394	-0.126 (-0.417, 0.165)	0.394	-0.125 (-0.274, 0.023)	0.098
Substituted by carbohydrates								
M1	-0.178 (-0.269, -0.087)	<0.001	-0.069 (-0.128, -0.010)	0.021	-0.119 (-0.378, 0.140)	0.368	-0.138 (-0.267, -0.009)	0.036
M2^b^	-0.042 (-0.119, 0.035)	0.287	0.028 (-0.019, 0.076)	0.236	-0.093 (-0.357, 0.171)	0.490	-0.115 (-0.244, 0.015)	0.084

Abbreviations: M1, Unadjusted model; M2^a^, Model adjusted for BMI (kg/m^2^), smoking (yes/no), and self-reported physical activity (inactive/active) in HUSK3; M2^b^, Model adjusted for BMI (kg/m^2^), smoking (yes/no), and self-reported physical activity (inactive/active) in HUSK2.

#### Longitudinal relationship of protein intake with ASMM and HGS

Unadjusted longitudinal analysis of dietary protein intake in HUSK2 and ASMM in HUSK3, showed a statistically significant decrease in ASMM when substituting 1E% of TP with fat or carbohydrates in men only. Additionally, substituting 1E% AP with fat or carbohydrates showed a statistically significant decrease in ASMM for both men and women, whereas substitution of AP with PP was only associated with a statistically significant ASMM decrease in men. Following adjustment for HUSK2 covariates, no longitudinal associations of substituting TP and AP remained significant in either sex. Unadjusted longitudinal analyses of protein intake in HUSK2 and HGS in HUSK3 showed no statistically significant associations between substitution of 1E% of TP with fat or carbohydrates or between substitution of 1E% AP with PP, fat, or carbohydrates in men. However, in women, substitution of 1E% TP with fat and substitution of 1E% AP with fat or carbohydrates showed a statistically significant decrease in HGS. Following adjustment for HUSK2 covariates, no associations between substitution of TP and AP were statistically significant in either sex.

#### Interpretation of estimates

For ASMM, the unadjusted β-coefficient of −0.090 in men and −0.111 in women obtained by substitution of TP with carbohydrates (Table 3) translates to a decrease of 90 g lean mass in men and 111 g lean mass in women for every 1E% of dietary protein that was substituted by carbohydrates. Although not statistically significant, for HGS, the unadjusted beta coefficient of −0.061 in men and −0.059 in women obtained by substitution of TP with carbohydrates (Table 3) translates to a decrease of 61 g in HGS in men and 59 g in HGS in women for every 1E% of dietary protein that is substituted by carbohydrates.

## Discussion

The current study investigated the cross-sectional and longitudinal associations of substituting amount and source of dietary protein with ASMM and HGS in community-dwelling older adults in Western Norway. Cross-sectional analyses revealed that substitution of TP and AP by fat and carbohydrates was negatively associated with ASMM in women but not in men, although no longitudinal associations were found between substitution of dietary protein intake and ASMM in either sex. Substitution of dietary protein intake was not associated with HGS in cross-sectional or longitudinal analyses in either sex.

The cross-sectional associations of TP and AP with ASMM (when substituted by fat or carbohydrates) among women in our study conform with the findings of a cross-sectional study by Geirsdottir et al. [31], in which protein intake was positively associated with muscle mass among community-dwelling older adults (>65 y) with a mean protein intake of >0.8 g/kg bw/d. Additionally, these findings are consistent with those of an analysis by Houston et al. [20] within the Health, Aging, and Body Composition (Health ABC) Study, which found a positive association between higher protein intake and preservation of ALM over a 3-y follow-up period in men and women. On the contrary, a longitudinal study in older Chinese adults [1] found that TP intake was not associated with longitudinal change in dual-energy X-ray absorptiometry (DXA)-measured total limb lean mass. The role of dietary protein intake in the maintenance of muscle mass and function has been widely researched. An adequate intake of dietary protein may exert positive effects by stimulating MPS and subsequently facilitating accretion of lean body mass [32]. Other mechanisms by which dietary protein intake is suggested to be beneficial for muscle mass and strength include mTOR pathway activation, enhanced mRNA translation, and greater levels of insulin-like growth factor 1, which are all linked with anabolic effects on muscle [33,34].

A range of experimental and observational studies have been conducted in an effort to identify strategies to prevent and delay the prominent declines in muscle mass, strength, and physical functioning seen with aging [35]. Although associations of TP and AP with ASMM in our study were not statistically significant in cross-sectional analyses for men or in longitudinal analyses for either sex, the predominantly negative beta-coefficients derived from substituting TP and AP with fat or carbohydrates may point toward a presumably negative impact of a reduced dietary protein intake on ASMM. The sex differences observed in cross-sectional analyses of TP and AP may partly be due to differences in the outcome variable, ASMM, for men and women. Although only ∼5% of men had values below the EWGSOP2 cut-off used to define low ASMM, as many as ∼37% of women exhibited low values for ASMM in our study population. This may reflect greater variation in ASMM for women than men and suggests a more critical role of dietary protein in the presence of low ASMM. A sex-specific analysis by Elstgeest et al. [36] within the Health ABC Study found an association between higher protein intake and less ALM decline over a 3-y follow-up period in women, whereas such an association was not evident in men. Elstgeest et al. [36] suggest that the finding of a sex-specific association of protein and ALM in their study may be attributed to the higher MPS rate in women than men, as reported in some studies [37,38]. Additionally, the authors highlight that older women experience an accelerated decline in muscle mass, suggesting a concurrent increase in muscle protein breakdown, and point to the upregulation of both stimulatory and inhibitory muscle growth-regulatory genes in postmenopausal women [36,39]. This may perhaps indicate a greater role of dietary protein intake in the preservation of muscle mass in older women than in men. Within our cohort, no cross-sectional or longitudinal associations were evident in either sex between TP or AP substitution and HGS. These findings are consistent with those of a pooled analysis of 4 longitudinal aging cohorts by Mendonça et al. [40], in which no associations were reported between baseline protein intake and HGS measured prospectively.

The evidence pertaining to substitution of AP with PP showed a lack of associations in cross-sectional and longitudinal analyses in both men and women. Furthermore, no consistent pattern was evident in the direction of the β-coefficients for the substitution of AP with PP. This may indicate that the source of protein is of less importance, particularly when the requirement for total energy intake and total protein intake is fulfilled. This finding is highly relevant in the context of the shift toward a predominantly plant-based diet, particularly encouraged by the recently published NNR 2023 [41]. There is a scarcity of studies investigating the distinct roles of PP and AP in relation to musculoskeletal health. However, a study investigating the role of protein sources on muscle mass in middle-aged and older Chinese adults concluded that higher dietary intakes of protein were associated with greater skeletal muscle index regardless of the ratio of AP-to-PP [42]. In similarity with our study population, the mean protein intake in the mentioned study was well above the current recommendation for protein intake of 0.8 g/kg bw/d [42].

The lack of associations between dietary protein intake and muscle mass and strength evident in the present study may be attributed to several factors. In the United States and Canada, the recommended daily allowance (RDA) for dietary protein intake for adults is 0.8 g/kg bw/d [43]. However, due to the presence of anabolic resistance in older adults, a higher RDA for dietary protein has been suggested to be beneficial for the maintenance of muscle mass and strength [44]. Based on the NNR 2012, the Norwegian Directorate of Health currently recommends an intake of >0.8 g/kg bw/d for adults aged 18-65 y, whereas older adults aged >65 y are recommended a daily intake of 1.1 to 1.3 g/kg bw/d. In our study population, the estimated mean daily protein intake was 1.16 g/kg bw/d in HUSK2 and 1.60 g/kg bw/d in HUSK3. A systematic review by Morton et al. [45] concluded that a protein intake exceeding the threshold of ∼1.6 g/kg bw/d did not exert additional RET-associated benefits to muscle mass and strength in healthy adults. Another study [44] among middle-aged US adults found significantly lower mean ALM in women belonging to the low-protein group (<0.8 g/kg bw/d) than the moderate protein group (≥0.8 to <1.2 g/kg bw/d), as well as a higher risk of low lean mass in men belonging to the low-protein group than the moderate protein group. However, no such differences were evident between the moderate and high (≥1.2 g/kg bw/d) protein groups, which may suggest that an intake exceeding a certain threshold is not associated with additional benefits to skeletal muscle mass in healthy individuals. As mentioned previously, low ASMM was evident in a higher proportion of women (36.6%) than men (5.2%) in our cohort. More importantly, the prevalence of men and women below the EWGSOP2 cut-offs for low HGS was 1.1% and 1.8%, respectively. Hence, lack of variation in both the exposure and outcome variables may possibly have led to no findings due to the ceiling effect.

Although the protein intake estimate of 1.6 g/kg bw/d in HUSK3 is higher than what has been reported in other comparable cohorts [46], it may possibly be attributed to overestimation by the WebFFQ, as the risk of overestimation is increased in FFQs that inquire about a very large number of items [[47], [48], [49]]. However, it may also, to an extent, reflect a true high intake, considering the likely presence of “healthy volunteer” selection bias in HUSK, as described in a previous study [50]. Notably, the estimated percentage of protein energy in HUSK3 (∼18 E%) was comparable with that of Norkost3, the third national dietary survey conducted among adults in Norway, which is intended to be representative of the general Norwegian population aged 18–70 y [51]. The quality of the dietary data in the current study is further substantiated by our previously conducted validation study, which found a correlation coefficient of 0.50 for protein, whereas cross-classification of protein intake quartiles revealed that 85% of the participants were categorized into the same or adjacent quartiles as with the reference method [28]. Although methodologic limitations may affect absolute estimates of protein intake, the acceptable ranking abilities of the WebFFQ indicate that participants with low and high intakes may be grouped separately, suggesting that its dietary intake estimates are still useful for the purpose of studying the effects of isocaloric substitution of protein [28].

The discrepancy between the findings in the current study and some previously conducted studies may partly be attributed to the use of isocaloric substitution models in the current study. Most previous studies have analyzed the isolated effect of protein intake on musculoskeletal outcomes. Isocaloric substitution allows the investigation of the effect of a specific nutrient while simultaneously accounting for the nutrients it is being substituted with, hence providing additional information that may be beneficial in terms of guiding future intervention studies [52]. Importantly, the substitution model considers that the isocaloric increase or decrease of one macronutrient inevitably leads to a change in the relative intakes of other macronutrients [53]. Although findings in the current study predominantly did not reach statistical significance, the substitution model provides valuable indications regarding the direction and potentially variable effects of replacing protein with alternative macronutrients.

Various RCTs have proposed that supplementation with dietary protein has beneficial effects on lean mass and muscle strength only when administered in combination with RET [54]. Physical activity (PA) has been established to be an important anabolic stimulus of muscle mass and should, thus, be considered when studying the relationship of protein intake with parameters of body composition and physical function. Assessment of PA in our study, unfortunately, did not include any specification of the types of PA that participants engaged in, and responses are inevitably affected by subjective interpretations of what constitutes PA. Hence, a limitation of the current study is the use of self-reported data on PA to categorize individuals as either physically active or inactive. The current study may, thus, have benefited from an objective assessment of PA. In addition to the amount and source of dietary protein, meal-time distribution of protein intake has been proposed to be of importance to skeletal muscle health [55]. Emerging evidence suggests that, particularly in older adults, maintaining a protein intake threshold of ∼25 to 30g/meal may benefit MPS stimulation and subsequent preservation of muscle mass and strength [56,57]. Unfortunately, meal-time distribution of protein intake was not considered in the current study.

Houston et al. [20] reported that the association found between protein intake and 3-y follow-up ALM was lost when excluding those who did not provide complete longitudinal ALM data. This may indicate that the initial association was driven by participants who were lost to final follow-up, who are more likely to be unhealthier than those completing the full study course. The presence of healthy volunteer bias likely represents a challenge in the current study because HUSK participants have been subjected to “double-positive” sampling in the sense that only participants who previously attended HUSK2 were reinvited to HUSK3, and those who died between the 2 study waves were lost to follow-up. Additionally, HUSK3 participation required subjects to be able to visit the study center, which inherently demands a certain level of functional ability. Hence, our findings may not be applicable to populations with limited functional capacity. It should also be noted that ASMM and HGS were only measured in HUSK3, making it impossible to determine the longitudinal association between dietary protein intake and changes in muscle mass and strength. Although the WebFFQ and PB-FFQ were similar in several aspects, the estimated energy intake being considerably higher in HUSK3 than HUSK2 indicates overestimation due to the WebFFQ being more comprehensive.

In recent years, it has been emphasized that the age-related loss of muscle mass and strength may initiate as early as middle age. Hence, a key strength of the current study includes the highly relevant follow-up time of ∼20 y between middle age and old age, which allowed the assessment of longitudinal associations. Although self-reported dietary intake data are known to be an error-prone exposure, the use of extensive and validated FFQs in both HUSK2 [[25], [26], [27]] and HUSK3 [28] lends credibility to the current study. The use of commonly applied methods for the assessment of muscle mass and muscle strength further strengthens the quality of the current study. The use of DXA and BIA has been shown to provide reliable ASMM estimates [5,58], and acceptable relative validity has been demonstrated for the method [[59], [60], [61]]. A recent systematic review [62] demonstrated that HGS was the most commonly applied measure for assessment of muscle strength in older people and concluded that HGS was associated with mobility, balance, and activities of daily living outcomes. [5]

We did not find substitution of amount and source of dietary protein intake to be associated with muscle mass and strength in the current study. Despite the general notion that older adults have increased needs for dietary protein intake, there is a scarcity of evidence that links low dietary protein intake in middle and older age with greater age-associated loss of muscle function [16]. Although protein requirements may be altered in individuals with sarcopenia, frailty, and/or malnutrition, current evidence does not clearly establish whether healthy adults may benefit from increasing their protein intake [16]. A recent review by Nishimura et al. [7] underscores that the forthcoming recommendations to increase dietary protein intake are based on acute feeding studies, which pose limitations when extrapolating the data to whole-body protein requirements. Importantly, a recent systematic review by Hengeveld et al. [63] concluded that there was “insufficiently convincing data” that increasing protein ≥0.8 g/kg bw/d exerted health benefits. There is consequently a need for more prospective longitudinal studies with repeated and reliable assessment of habitual dietary intake as well as concurrent measures of muscle mass and function. Although the current study cannot establish causal relationships of dietary protein intake with muscle mass and strength, we recon it as a valuable contribution to the knowledge on protein intake adequacy in older adults with high-functional capacity which may, thus, have potential implications for policymakers establishing dietary recommendations.

## Authors contributions

The authors’ responsibilities were as follows – ZS, HRR, AH, JD: designed the study; ZS: was involved in the collection of dietary intake data in HUSK3; ZS: responsible for data entry, statistical analysis, and writing the manuscript for the current study; HRR: primary responsibility for the final content; all authors: contributed to the interpretation of the findings; all authors: read and approved the final manuscript.

## Funding

The current study was funded by the Mohn Nutrition Research Laboratory. The funders had no role in the study design, collection, analysis, and interpretation of data or writing of this article.

## Data availability

Data used in the current study are available from the corresponding author upon request.

## Conflict of interest

The authors report no conflicts of interest.
