# Supplementary material for: The Association of Isocaloric Substitution of Dietary Protein in Middle Age with Muscle Mass and Strength in Old Age: The Hordaland Health Study
Source: Curr Dev Nutr. 2023 Nov 30;8(1):102052. doi: 10.1016/j.cdnut.2023.102052 (PMC10770717; doi:10.1016/j.cdnut.2023.102052)
Supplement: Multimedia component 1 [file mmc1.docx]

# **Online Supplementary Information** *Current Developments in Nutrition*

**The association of isocaloric substitution of dietary protein in middle age with muscle mass and strength in old age: The Hordaland Health Study**

Zoya Sabir^1, *^, Jutta Dierkes^1^, Anette Hjartåker^2^, Hanne Rosendahl-Riise^1^
^1^ Centre for Nutrition, Mohn Nutrition Research Laboratory, Department of Clinical Medicine, University of Bergen, Norway
^2^ Department of Nutrition, Institute of Basic Medical Sciences, University of Oslo, Norway

**^*^ Correspondence to first author:** Zoya Sabir, [zoya.sabir@uib.no](mailto:zoya.sabir@uib.no), Department of Clinical Medicine, 5020 Bergen, Norway. ORCID: 0000-0003-0744-5611

**Supplementary Table 1:** Characteristics of participants in the second (HUSK2) and third (HUSK3) wave of the Hordaland Health Studies

| Characteristic^a^ | HUSK2  All (*n*=2060) | HUSK3  All (*n*=2060) | p-value^b^ |
| --- | --- | --- | --- |
| Height (m) | 172 (9.0) | 171 (9.2) | <0.001 |
| Weight (kg) | 75.1 (13.5) | 77.2 (14.5) | <0.001 |
| BMI (kg/m^2^) | 25.3 (3.5) | 26.4 (4.1) | <0.001 |
| Waist (cm) | 85.4 (11.2) | 95.2 (12.6) | 0.000 |
| Total body fat mass (kg)^c^ | 22.3 (8.9) | 27.3 (8.8) | <0.001 |
| Total body fat mass percentage (%)^c^ | 31.2 (9.3) | 35.3 (8.3) | <0.001 |
| Total body lean mass (kg)^c^ | 48.5 (11.0) | 49.9 (10.9) | <0.001 |
| Total body lean mass percentage (%)^c^ | 68.8 (9.3) | 64.7 (8.3) | <0.001 |
| Regular, current smoking^d^ | 31.5% | 7.8% | <0.001 |
| Leisure time moderate physical activity^d^  *≥3 hours per week* | 47.6% | 70.2% | <0.001 |
| Leisure time hard physical activity^d^  *Any* | 77.7% | 86.9% | <0.001 |

BMI, body mass index. Slight deviations in *n* for some participant characteristics due to lack of data.
^a^ Continuous variables are presented as mean±standard deviation. Categorical variables are presented as percentages
^b^ p-value for difference between characteristics in HUSK2 and HUSK3. Significance level p<0.05
^c^ Measured by dual-energy X-ray absorptiometry in HUSK2 and by bioelectrical impedance analysis in HUSK3
^d^ Based on self-reported data
